# An Investigation of Factors Affecting Bowel Movement Hygiene in Autistic Children

**DOI:** 10.1155/oti/5464138

**Published:** 2026-03-28

**Authors:** Nadine Stacey, Beverley Turtle, Jean Daly-Lynn

**Affiliations:** ^1^ School of Health Sciences, Ulster University, Belfast Campus, Belfast, UK, ulster.ac.uk

**Keywords:** autism, bottom wiping, occupational therapy

## Abstract

Bowel movement hygiene is an essential life skill. Autistic children can have difficulties attaining this skill. There is limited research exploring postbowel movement hygiene (PBMH) and autism. This study explores factors of PBMH for autistic children. An online survey was developed, included open and closed questions, and distributed via Facebook groups. It was completed by *N* = 74 parents. Descriptive statistics and thematic analysis were used to analyze the data. Statistically significant correlations were found between variables related to the sensory and motor aspects of PBMH, scared of sitting on toilet and refusal to engage in PBMH (*p* ≤ 0.5). Four qualitative themes were identified: toilet paper, difficulties in reaching the bottom, health related factors, and successful strategies used by parents. Future research is needed to strengthen the validity of these findings and produce practice guidelines for occupational therapists working in this area.

**Plain Language Summary**

There are very few studies reporting on how hard it is for autistic children to learn to wipe their bottom. This paper reports on the findings of an online survey of parents’ thoughts on their autistic child learning to wipe their bottom. Seventy‐four parents completed the survey. Problems were identified including difficulty reaching bottom and tearing toilet paper. Sensory issues were described including difficulties in seeing, touching, or smelling stool. Parents offered successful ways that have helped their child as well as explaining why health‐related issues have made this skill difficult to learn. The findings can be used to support occupational therapists and other therapists in helping autistic children learn how to wipe their bottom.

## 1. Introduction

Autistic spectrum disorder (ASD) is a diverse and lifelong developmental disability which affects how people communicate and interact with the world and includes atypical patterns of activities and behaviors [1]. The Diagnostic and Statistical Manual of Mental Disorders—Fifth Edition (DSM‐5 [2]) describes features of the condition including severe impairment in reciprocal social interaction and communication skills, a restricted range of interests and atypical sensory reactivity. Statistics relating to the prevalence of autism range from 1 in every 100 children [3] to 1 in every 36 children [4].

Children with ASD have functional difficulties in fine and gross motor skills including motor planning, motor coordination, postural stability, functional balance and praxis [5–7]. Praxis reflects the ability to organize actions, body movement and sequencing, and has been linked to daily functions and activity level [8]. There is a growing body of evidence indicating that difficulties in praxis are common among people with ASD and are a key trait [9–12]. Mailloux et al. [13] suggest that sensory‐based motor disorders interfere with daily routines including self‐care.

It is estimated that more than 80% of children with ASD have sensory processing difficulties [14]. Hyperreactiviy or hyporeactivity to sensory input is now a diagnostic criterion for ASD in the Diagnostic and Statistical Manual of Mental Disorders—Fifth Edition (DSM‐5 [2]). Atypical sensory reactivity is described as over or under responsivity to sensory stimuli [8]. Over‐reactivity can present with negative reactions to sensory stimuli including touch, smell, and sound [15]. Individuals with under‐reactivity may be unaware or nonresponsive to sensory stimuli in the environment [16]. Sensory processing problems in ASD can have a detrimental effect on daily functioning performance [17–19].

According to Jasmine et al. [20], children with ASD have severe difficulties in daily living skills, including toileting, due to atypical sensory responses and poor gross and fine motor skills. Acquiring independence in toileting abilities is considered an important milestone of childhood; in the typically developing population, these skills are usually acquired by the age of four [21–23]. Individuals with developmental disabilities such as ASD are more likely to experience toileting problems than the general population [24] and toileting opposition affects almost half of children with ASD [25]. In a study by Koshy [26] 68% of children with ASD and sensory processing difficulties also had functional problems with their toileting skills. Independent toileting allows individuals to participate fully in school programs and extracurricular activities, community‐based living as well as fostering a sense of autonomy and self‐esteem [27, 28]. Issues in this area can limit an individual′s overall health, independence, and social participation [29, 30]. Parents of autistic children rank toileting as one of the most stressful tasks [28, 31, 32].

Occupational therapy (OT) is concerned with understanding and supporting the everyday activities in which people engage [33, 34]. Bowel management constitutes one important activity of daily living [35]. A common toileting protocol developed by Azrin and Foxx [36] and later adapted by Leblanc et al. [37] was specifically aimed at toilet training individuals with ASD. Two systematic reviews examined studies that used these protocols to review the effectiveness of toileting skill acquisition for individuals with ASD [38, 39]. Simon et al. [39] used the operational definition, for the purposes of their study, that independent toileting included cleaning oneself afterwards. Despite this, no reference was made to postbowel movement hygiene (PBMH) throughout the review, instead referring to components such as hand washing or dressing. Saral and Burcu [38] identified steps in a toileting chain including appropriate use of toilet paper. When reporting on the acquisition of skills across the 23 studies, improvement in splinter skills such as sitting on the toilet and flushing were reported, but no reference was made to PBMH. Stokes et al. [40] reported that 86% of 30‐surveyed males with ASD could not effectively conduct their own PBMH. In a study by Bailey et al. [41], that looked at skill attainment among adults with fragile X syndrome, found that only 66% of men could complete PBMH independently, whilst this figure rose to 93% in females. Bailey [41] identified the need to use a toileting subscale to examine where the breakdown in attainment arose and included “wipes independently” in the success criteria.

Beaudry‐Bellefeuille et al. [42, 43] examined sensory behaviors in their Toileting Habit Profile Questionnaire, which includes a test item where parents are asked to score “*my child refuses to wipe or be wiped after pooping*”. However, research using this tool with children with ASD is limited and no specific correlations between sensory reactivity and PBMH have been made. The Profile of Toileting Issues screening tool was developed to identify toileting problems in individuals with intellectual difficulties including ASD [44]. The tool identifies motor difficulties, fear and smears in underwear as interfering with toileting, but PBMH is not included as a screening question.

Only two previously published papers specifically focus on PBMH for individuals with ASD. Stokes et al. [40] describe success using a 10‐step correspondence training to teach three adult males with ASD to effectively complete PBMH. Byra et al. [45] reported success in using a doll simulation to teach two children with ASD to conduct PBMH. Both studies were conducted with a very small sample size and with individuals who possessed good motor and verbal skills. Neither addresses the underlying reasons as to why the individuals initially found this skill difficult.

Mac Alister [46] postulates in a nursing practice review paper that difficulties arising in the acquisition of toileting skills for children with ASD may be in relation to sensory differences and states wiping to be a common difficulty. Coucouvanis [47] identified routine, communication, social interaction, sensory stimulation, learning profile, and biological functions as challenges to PBMH. Wheeler [48] adds sensory sensitivities, sensory awareness, motor planning, and anxiety to this list, with factors including: physiological, motor impairments, communication both expressive and receptive, social awareness, sensory preferences, and psychological wellbeing identified by Simon et al. [39].

From the literature, there is evidence that children with ASD find functional everyday skills, including toileting, difficult and that independent toileting is an important milestone. There is a paucity of evidence to the reasons why individuals with ASD find specific steps in toileting challenging; there is very limited evidence looking at PBMH specifically. As Francis et al. [49] highlighted, the function of toilet behaviors, including PBMH, needs to be identified to target intervention programs. Due to the lack of current literature on the subject, there is a need to add to the body of evidence to support the acquisition of PBMH in this client group. OT is a profession well‐placed to develop toilet training interventions, but an increased understanding of the reasons behind PBMH difficulties for children with ASD is needed to inform evidence‐based interventions.

The purpose of this study was to identify key factors and barriers in completing PBMH for children with ASD as identified by parents. It additionally seeks to suggest target intervention areas for occupational therapists and other clinicians working in this area.

## 2. Method

### 2.1. Design

The study was a survey design that collected quantitative data in addition to a qualitative response from one question. Saral and Burcu [38] identified the need for research to evaluate the perception of toilet training components from a parental perspective. This study invited parents to answer questions around their experience of supporting an autistic child to learn PBMH. The survey was developed to reflect broad themes in the literature including sensory reactivity, motor skills, environmental demands, stress, and anxiety (Table 1). The survey design was supported by engagement with four parents who piloted the survey, all of whom have an autistic child, with ages ranging from 7 to16. The piloting stage resulted in minor changes to the wording of three of the survey questions. The questions of the survey were assigned a score in line with the responses of never, occasionally, frequently, and always (1–4); easily, with some difficulty, and with lots of difficulty (1–3); and no help, supervision, verbal instruction, and physical help. Higher scores were indicative of greater difficulty with PMBH. Ethical approval was provided by the Ulster University Nursing and Health Sciences Research Filter Committee (Project number: FCNUR‐23‐081).

**Table 1:** Survey Questions.

Question	Response options
Do you have any cultural beliefs around toileting?	Yes/no
If you answered yes to the above question, would you feel comfortable to briefly explain these?	
My child shows a strong reaction at the sight of his/her poo	Never, occasionally, frequently, always
My child shows a strong reaction to the smell of his/her poo	Never, occasionally, frequently, always
My child shows a strong reaction if they touch poo with their hands	Never, occasionally, frequently, always
My child finds it difficult to maintain a sitting balance on the toilet	Never, occasionally, frequently, always
My child is scared to sit on the toilet	Never, occasionally, frequently, always
My child can tear and fold toilet roll/toilet paper	Easily, With some difficulty, With lots of difficulty
My child finds it difficult to reach and locate their bottom accurately for wiping	Never, occasionally, frequently, always
My child refuses to wipe their bottom	Never, occasionally, frequently, always
My child has soiled underwear	Never, Occasionally (monthly), Frequently (weekly), Always (daily)
To successfully complete bottom wiping my child needs	No help, Supervision, Verbal instruction, Physical help
Does your child request or prefer to poo whilst wearing a nappy/pull‐up/pad?	Never, occasionally, frequently, always
Does your child avoid doing a poo outside of their home environment?	Yes/no
Has your child ever smeared or played with their own poo?	Never, Frequently (at least once a week), Has done in the past (at what age and how long), Other
My child has received support from a professional in the acquisition of bottom wiping	No support, Occupational therapist, Teacher, Physiotherapist, Other (please specify)
How stressful do you find the issue of bottom wiping acquisition for your child?	Rating scale: 1=No stress at all to 10= Highly stressful
Is there anything that is not captured in the questions above that you would like to add regarding your child′s ability to complete bottom wiping?	

### 2.2. Participants

Participants were recruited using digital snowball sampling via Facebook. Recent systematic reviews summarized Facebook as being a useful and successful recruitment tool in health care, with improved participant uptake, cost effectiveness, recruitment of samples in a relatively brief time, snowball recruitment opportunities and improved participant uptake in hard‐to‐reach demographics [50–52]. Inclusion criteria for the study were parents of children with ASD who range in age from 4 to 18 years. The lowest age of 4 years was chosen as the recognized age in which PBMH on average is achieved in the typical population [21–23].

Although participants volunteered to undertake the survey, it is possible they may have had an unpleasant experience in relation to toileting in the past and become distressed whilst completing the survey. Hence, participants were signposted to organizations that support in toileting such as The Children’s Bowel and Bladder Trust (ERIC) and The National Autistic Society, at the start of the survey and again at the end of the survey as part of the submission statement. The chief investigators contact details were also provided in the participant information sheet so they can be contacted with any concerns.

### 2.3. Data Collection

Data were collected using an online survey tool, completed by parents of children with ASD. Participants completed the survey anonymously through the online platform Microsoft Forms. An advert was produced that briefly explained the study and included a link and QR code to access the participant information sheet, consent form and survey. This was posted on Facebook pages relating to autism. For the purposes of the study, the researcher created a Facebook page and profile for the sole use of this research. Facebook groups relating to autism were contacted via the moderators to ask for permission to join and/or post the research advert. These were found using the Facebook search function using the words “autism”/“ASD” and parent(s). Groups were disregarded if they had less than 1000 members. Data sampling lasted for 12 weeks to allow for snowball recruitment, often common in Facebook participant recruitment [53].

### 2.4. Data Analysis

The Statistical Package for the Social Sciences (SPSS) Version 29 (IBM Corp.) was used for all statistical analyses. Descriptive statistics were completed for demographic details and scores across survey responses. Based on the challenges to PBMH [39, 47, 48], associations between survey questions related to sensory, balance, fine motor, gross motor, fear, soiled underwear, and refusal to wipe were examined using Spearman′s rho and interpreted as small for correlations < 0.3, medium for scores between 0.3 and 0.5, and for scores > 0.5 [54].

Inductive content analysis was used to analyze the qualitative data generated from the open‐ended question in the survey. Inductive content analysis is described as a good type of analysis to use when there is scarcity in existing research in the topic field [55, 56] and particularly suitable when the researcher is aiming for a practical answer or application of the findings, such as to develop intervention recommendations [57]. The qualitative data was downloaded into word format and read multiple times for the researchers to become familiar with the text, an important step to ensure comprehension [57]. Meaning units were selected from each participant response and condensed by removing repetition and words not meaningful to the research aims [58]. To illustrate this point, responses such as “*My son is ASD with PDA profile*”, were removed as they did not add meaning to the subject being researched. First round coding was used to identify big picture meaning and initial categories, with second round coding identifying subcategories.

## 3. Results

### 3.1. Demographic Information

The survey was completed by 77 participants (Table 2). Three responses were deleted due to one participant not indicating consent, one participant not indicating prior reading of the participant information sheet, and one child in the survey being above age criteria.

**Table 2:** Characteristics/participant demographics *n* = 74.

Child sex	
Female	57
Male	17
Child age	
Mean	9.2162
Standard deviation	0.42124
Minimum	4
Maximum	18
Parental gender	
Female	72
Male	0
Other	2
Cultural beliefs relating to toileting	
Yes	0
No	74

Spearman′s rho correlations between PBMH variables are shown in Table 3. Small significant correlations were found between being scared to sit on the toilet and frequency of soiled underwear; managing toilet roll and locating bottom; managing toilet roll and refusal to wipe; locating bottom and soiled underwear; refusal to wipe and preference to open bowels in nappy/pad/pull‐up.

**Table 3:** Spearman′s rho correlation coefficients for association between 11 toileting variables.

Variables		Sight	Smell	Touch	Balance	Fear	Toilet roll	Locating bottom	Refusal to wipe	Soiled underwear	Help	Bowel movement in nappy
Sight	Correlation coefficient	1.000	0.646 ^∗∗^	0.414 ^∗^	0.078	0.375 ^∗∗^	0.142	−0.060	−0.011	0.164	0.057	0.165
	N	74	74	74	74	74	74	74	72	73	74	74
Smell	Correlation coefficient	0.646 ^∗∗^	1.000	0.516 ^∗∗^	0.188	0.308 ^∗∗^	0.131	−0.036	0.121	0.005	−0.188	0.008
	N	74	74.	74	74	74	74	74	72	73	74	74
Touch	Correlation coefficient	0.414 ^∗∗^	0.516 ^∗∗^	1.000	0.135	0.137	−0.053	−0.130	−0.012	0.009	−0.207	−0.116
	N	74	74	74	74	74	74	74	72	73	74	74
Balance	Correlation coefficient	0.078	0.188	0.135	1.000	0.225	,035	0.027	0.075	0.085	−0.098	0.030
	N	74	74	74	74	74	74	74	72	73	74	74
Fear	Correlation coefficient	0.373 ^∗∗^	0.308 ^∗∗^	0.137	0.225	1.000	0.204	−0.021	0.038	0.294 ^∗^	0.030	0.418 ^∗∗^
	N	74	74	74	74	74	74	74	72	73	74	74
Toilet roll	Correlation coefficient	0.143	0.131	−0.053	0.035	0.204	1.000	0.295 ^∗^	0.262 ^∗∗^	0.205	0.091	0.349 ^∗∗^
	N	74	74	74	74	74	74	74	72	73	74	74
Locating bottom	Correlation coefficient	−0.060	−0.036	−0.130	0.027	−0.021	0.295 ^∗^	1.000	0.397 ^∗∗^	0.288 ^∗^	0.420 ^∗∗^	0.353 ^∗∗^
	N	74	74	74	74	74	74	74	72	73	74	74
Refusal to wipe	Correlation coefficient	−0.011	0.121	−0.012	0.075	0.038	0.262 ^∗^	0.397 ^∗∗^	1.000	0.045	0.345 ^∗∗^	0.252 ^∗^
	N	72	72	72	72	72	72	72	72	71	73	72
Soiled underwear	Correlation coefficient	0.164	0.005	0.009	0.085	0.294 ^∗^	0.205	0.288 ^∗^	0.045	1.000	0.175	0.375 ^∗∗^
	N	73	73	73	73	73	73	73	71	73	73	73
Help	Correlation coefficient	−0.057	−0.188	−0.207	−0.098	0.030	0.091	0.420 ^∗∗^	0.345 ^∗∗^	0.175	1.000	0.180
	N	74	74	74	74	74	74	74	72	73	74	74
Bowel movement in nappy	Correlation coefficient	0.165	0.008	−0.116	0.030	0.418 ^∗∗^	0.349 ^∗∗^	0.353 ^∗∗^	0.252 ^∗^	0.375 ^∗∗^	0.180	1.000
	N	74	74	74	74	74	74	74	72	73	74	74

*Note:* Spearman′s rho correlation coefficient: Single asterisk ( ^∗^) denotes correlation is significant at 0.05 level. Double asterisks ( ^∗∗^) denote correlation is significant at 0.01 level.

Moderate significant correlations (rho = 0.3–0.5, *p* ≤ 0.05) were found between having a strong reaction to the sight of stool and having a strong reaction if they touch the stool; a strong reaction to the sight of stool and scared to sit; strong reaction to the smell of stool and scared to sit; scared to sit and preferring to use a nappy/pad/pull‐up for bowel movement; difficulty with locating bottom and refusal to wipe; locating bottom and requiring help; locating bottom and preferring to use a nappy/pad/pull‐up for bowel movement; refusal to wipe and requiring help; soiled underwear and preferring to use a nappy/pad/pull‐up for bowel movement; and managing toilet roll and preferring to use a nappy/pad/pull‐up for bowel movement.

Strong significant correlations (rho > 0.5, *p* ≤ 0.05) were found between a strong reaction to the sight of stool and the smell of stool, and a strong reaction to the touch and smell of stool.

Parents were asked to rate how stressful they find the issue of bottom wiping acquisition for their child on a scale of 1–10, with a score of 10 indicating a high level of stress. The mean score was 5.88 (SD = 2.62); 11% of participants gave this the highest score of 10, and 68% of participants gave this a score of 5 or above, indicating some level of stress for the acquisition of bottom wiping by their child. Thirteen of the 74 participants reported their child had received professional help for PBMH: seven participants had received help from an occupational therapist; three from a nurse; one from a health visitor; one from a physiotherapist; and one from a teacher.

The qualitative, open‐ended question was completed by 43 parents out of the 74. Following inductive content analysis, as described in the data analysis section of this paper, five distinct themes were developed from the data (Table 4).

**Table 4:** Qualitative themes.

Theme number	Theme	Subthemes
1.	Issues relating to toilet paper and PBMH	Use of wet wipes, Textural difficulties with toilet paper, Use of excessive amounts of toilet paper, Blocking toilet due excessive amounts of toilet paper being used, Difficulties in relation to different toilet paper dispensers outside of the home environment.
2.	The reasons why difficulties exist in reaching the bottom for PBMH	Coexisting diagnoses
3.	Health related factors effecting PBMH	Constipation and use of laxatives, Existing health conditions, for example, irritable bowel syndrome
4.	Strategies to successful PBMH	Environmental adaptations, Cognitive strategies
5.	PBMH outside of the home environment	Refusal to open bowels outside of home environment

#### 3.1.1. Theme 1—Issues Relating to Toilet Paper

One of the most described challenges to PBMH was toilet paper issues. It was reported that using wet wipes created better success, that children did not like the texture of toilet paper, that wiping with standard toilet tissue made children sore, children chose to shower instead of wiping, and that different toilet tissue dispensers in the community were hard to use. It was also common that too much toilet paper was used, and subsequent blocking of the toilet occurred.

“She still won′t use toilet paper. We have a bin next to the toilet and she only wipes a poo with toddler wet wipes. These have to go away on holiday with us. I have never established if this is a habit, but she has always expressed that this is the most comfortable thing for her and became agitated at the suggestion she should use toilet paper. We haven′t broached this for years”. (Participant ID #53)

“A lot of my child’s improved progress with wiping his bottom appeared to be when he started to use flushable toilet wipes. He expressed the toilet paper made his bottom sore and that because of this he could not clean himself easily”. (Participant ID #5)

#### 3.1.2. Theme 2—Issues Relating to Reaching the Bottom

One of the survey questions asked if there was difficulty for their child in locating and reaching the bottom for wiping. Some participants noted the reasons why reaching the bottom was motorically difficult, including coexisting diagnoses of developmental coordination disorder, obesity, poor balance, and a lack of understanding of verbal motor directions.

“My son has developmental coordination disorder, a condition often associated with autism. Because of this he struggles to physically reach around and make the wiping motion as his motor skills and hand eye coordination are poor”. (Participant ID #9)

“My child will not wipe her bottom from the back. She reaches it from the front and wipe towards the front! I tried to physically guided her hand to the back but it feels as if she can′t reach there?! I tried to show her lifting one bum off the seat too reach from under but she can′t do that due to her balance. This is a pain of my life and I didn′t even know it′s a thing with autistic kids!” (Participant ID #25)

#### 3.1.3. Theme 3—Health Related Factors

Managing gastrointestinal and other health conditions relating to toileting was described as a barrier for independent PBMH. This included constipation, use of laxatives, irritable bowel syndrome, hemorrhoids, and worms.

“It has been only recently since we started him on laxatives and he has started to recognize when he needs the toilet, leading to no accidents, that we have been able to start teaching him how to wipe”. (Participant ID #50)

#### 3.1.4. Theme 4—Strategies to Successful PBMH

Parents shared information on strategies that they have found useful in successful PBMH. This fell into distinct subcategories of environmental adaptations and cognitive strategies. Environmental adaptations included the use of different toilet seats or toilet seat adaptations.

“Will only sit on bathroom toilet using a folding travel seat. He won′t sit on the normal seat as afraid of falling in. This can all affect ability to wipe his bottom independently.” (Participant ID #4)

Cognitive strategies were rules, routine based learning, and use of social narratives.

“Difficult to begin with but once rules were made it’s easier‐ 4 pieces of toilet paper folded in a certain why and then 4 again‐ took a lot of practice but by following the ‘poo rules’ we’ve managed”. (Participant ID #66)

#### 3.1.5. Theme 5—Refusal to Open Bowels Outside of Home Environment

This theme relates indirectly to PBMH, and was highlighted as important in the data by parents. For many children, conducting bowel movements outside of their home environment is a source of anxiety, leading to secondary health problems such as fecal withholding or skin irritation caused by heavily soiled underwear that is not cleaned until they are in their home environment. This could then lead to secondary problems with PBMH.

“He will refuse to poo anywhere other than at home and if he needs to, and tries in a strange toilet, he will always come back out and say it’s gone away”. (Participant ID #15)

“She wouldn’t go outside of the home environment and sometimes not go at all and then would mess herself”. (Participant ID #46)

## 4. Discussion

This is the first study that directly addresses the reasons children with ASD may find the PBMH element of toileting difficult. Survey results suggest this can be attributed to sensory and motor factors. Results highlight that differing issues related to toilet paper and gastrointestinal health contribute towards the success or failure to conduct PBMH. Parents find supporting their child in this self‐care activity stressful and professional support is not often received. Whilst reviews of toilet training interventions with children with ASD can be found, none conduct a task analysis detailed enough to capture specific toileting skills such as PBMH.

Findings from the study suggest that sensory over‐reactivity to the sight, smell and touch of stool, creates a barrier to success in PBMH. According to the literature, 80% of children with ASD have sensory processing difficulties and 68% of children with ASD and sensory processing difficulties have functional problems with their occupational performance in daily activities including toileting [14, 26]. Radford and Anderson [59] identified that fecal incontinence, or soiling is often related to anxiety caused by sensory sensitivities and Beaudry‐Bellefeuille et al. [42, 43] identified 17 items relating to sensory responses in their Toileting Habit Profile Questionnaire. Studies have shown success in OT intervention for children with defecation disorders when consideration is given to the presenting sensory issues [60, 61]. This suggests that more attention should be given to the presenting sensory issues specific to PBMH.

The motoric elements of tearing and/or folding toilet paper and locating the bottom were highlighted in the findings from the data and suggest that they are key elements to the success of PBMH. As identified by Fournier et al. [5]; Memari et al. [6]; and Ming et al. [7], children with ASD have difficulties with their fine and gross motor skills, with a growing body of authors declaring these difficulties in terms of praxis [9–12]. Children without the prerequisite gross and fine motor skills needed for aspects of PBMH cannot be expected to be successful in this area, suggesting that toileting intervention from professionals needs to focus more on the development of the motor skills required for this task. In 2019, a teacher went viral when she posted a video of children learning to reach to wipe between two balloons tied on the back of the chair. Arguello et al. [29] supported the use of such simulated materials to practice the skills outside of naturally occurring opportunities; interventions of this nature would be supported by the findings of this research.

A theme identified from the qualitative data was the increased success in PBMH when using wet wipes instead of toilet paper. The exact mechanisms of how wet wipes are manipulated compared with toilet paper were not discussed in the qualitative data, but with most wipes being easily dispensed by pulling upwards on a wipe protruding from packaging, one can surmise that this negates the difficulties with the motoric elements of toilet paper use and could be considered an alternative technique to support PBMH in children with ASD. Tactile sensory preferences could also account for this difference; 60% of children with ASD display altered tactile sensitivity [62], including sensory over‐reactivity and sensory under‐reactivity. Beaudry‐Bellefeuille et al. [42, 43] identified many aspects of sensory reactivity in the toileting process. One could speculate that individuals are showing sensory over‐responsivity to the texture of toilet paper or alternatively are sensory under‐reactive and therefore it is easier to register a cold wet against the skin. For professionals going forward, it is important to establish sensory preferences through a standardized sensory assessment to best inform on supporting sensory strategies when conducting PBMH.

Findings from this study suggest that parents find the issues of their child′s PBMH stressful. Parents of children with ASD report eating and toileting as the hardest areas to manage and they are unsure about how they should be approached [63, 64]. Parental stress is higher in parents of incontinent children with disabilities and in children with bladder and bowel dysfunction than children who are toiled trained. Toileting independence prevents exclusion from social environments, reduces negative health outcomes, improves personal hygiene, prevents peer rejection [65] and has been linked to psychological benefits [39] but delays in these areas contribute to increased parental stress [66]. With parents playing such an important part in coregulating their child′s emotions as well as helping in self‐care activities, [67–69], interventions addressing parental stress and their own self‐care may be beneficial.

### 4.1. Limitations

The survey did not ask for full demographic information of the participants. Many children with ASD have co‐concurring difficulties with intellect or a dual diagnosis. Recent studies report that 30%–40% of children with ASD have an intellectual disability ([4, 70]) and studies report on intellect being the greatest indicator of functional outcomes [71]. This survey did not ask participants to indicate if their child had an intellectual disability or a dual diagnosis, which could affect their levels of independence in toileting and PBMH. It is known that more UK based Facebook groups gave permission for the study to be advertised than non‐UK based groups; as a result, the sample is not representative of the general population. In duplicating the posts on multiple ASD groups, Facebook temporarily suspended the account suspecting “suspicious” activity which limited its reach to more groups.

The research team did include a question relating to the auditory aspects of toileting such as reaction to the noise of a flushing toilet. As highlighted by Mayes et al. [72], many children with ASD have an unusual fear of toilets, which this research failed to address and could directly affect PBMH. As shown in the qualitative data, participants shared the reasons why locating the bottom was difficult for their child and strategies that they have found useful in successful PBMH. Survey questions related to these two areas could be included if the study was to be repeated. The survey covered a large age range (4–18); there is a significant difference in childhood development between the everyday skills of a 4‐year‐old and an 18‐year‐old, which was not examined in this study.

### 4.2. Recommendations for Future Research

The findings from the current study provide the basis of future work enabling a more focused approach to this topic area. An intervention to support PBMH could be developed as an outcome of these findings and evaluated. Specific information such as child age banding and additional diagnoses could be advantageous if the survey were repeated. Extra survey questions relating to the auditory aspects of toileting, reasons why reaching the bottom is difficult, and strategies that parents have found useful could be beneficial to include.

## 5. Conclusion

This study provides a greater understanding of the challenges faced in PBMH for autistic children and adds to the limited research in this area. This study identified areas that may affect successful PBMH in autistic children. The study suggests some target intervention areas for Occupational Therapists and other clinicians working in this area to improve independence in this essential life skill. Future research is needed to strengthen the validity of these findings and produce recommendations to support practice guidelines.

## Funding

No funding was received for this manuscript.

## Ethics Statement

Ethical approval was provided by the Ulster University Nursing and Health Sciences Research Filter committee (Project Number: FCNUR‐23‐081). All participants provided written informed consent.

## Conflicts of Interest

The authors declare no conflicts of interest.

## Data Availability

Data cannot be shared publicly because of ethical restrictions on sharing sensitive data. Access to the data can be provided following a successful application to Ulster University′s Nursing and Health Research Ethics Filter Committee. Ulster University′s Research Portal contains metadata on the dataset and instructions on how to request access to this dataset. This information can be accessed at (DOI under development).
